# Retraction Note: Functionalized graphene oxide nanosheets with folic acid and silk fibroin as a novel nanobiocomposite for biomedical applications

**DOI:** 10.1038/s41598-024-84474-1

**Published:** 2025-01-06

**Authors:** Reza Eivazzadeh-Keihan, Farkhondeh Alimirzaloo, Hooman Aghamirza Moghim Aliabadi, Ehsan Bahojb Noruzi, Ali Reza Akbarzadeh, Ali Maleki, Hamid Madanchi, Mohammad Mahdavi

**Affiliations:** 1 Catalysts and Organic Synthesis Research Laboratory, Department of Chemistry, Iran University of Science and Technology, 16846-13114 Tehran, Iran; 2 Protein Chemistry Laboratory, Department of Medical Biotechnology, Biotechnology Research Center, Pasteur Institute of Iran, Tehran, Iran; 3 Advanced Chemical Studies Lab, Department of Chemistry, K. N. Toosi University of Technology, Tehran, Iran; 4 Department of Inorganic Chemistry, Faculty of Chemistry, University of Tabriz, Tabriz, Iran; 5 Department of Chemistry, Iran University of Science and Technology, 16846-13114 Tehran, Iran; 6 Department of Biotechnology, School of Medicine, Semnan University of Medical Sciences, Semnan, Iran; 7 Drug Design and Bioinformatics Unit, Department of Medical Biotechnology, Biotechnology Research Center, Pasteur Institute of Iran, Tehran, Iran; 8 Endocrinology and Metabolism Research Center, Endocrinology and Metabolism Clinical Sciences Institute, Tehran University of Medical Sciences, Tehran, Iran

Retraction of: *Scientific Reports* 10.1038/s41598-022-10212-0, published online 13 April 2022

Editors have retracted this Article.

After publication of this Article, concerns about the data were brought to the attention of the Editors. Specifically:

- The image panels in Figs. 7c and 7d appear to overlap. These panels also appear to overlap with images published in an earlier paper with common authors [1], where they were described differently.
- The well images included in Fig. 8 appear to duplicate images published in an earlier paper with common authors [2], where they are described differently.

The Editors requested that the Authors provide full raw data and copies of their ethics approval and protocol for the use of blood samples drawn from a human donor, but the Authors were not able to do so. The Editors no longer have confidence in the reliability of the results and findings presented in this Article.

Hamid Madanchi disagrees with this retraction. The Editors were not able to obtain a current contact email for Farkhondeh Alimirzaloo. The remaining authors did not respond to correspondence from the Editors regarding this retraction.
